# Supplementary material for: A Hyper-Attenuated Variant of Rift Valley Fever Virus Generated by a Mutagenic Drug (Favipiravir) Unveils Potential Virulence Markers
Source: Front Microbiol. 2021 Feb 9;11:621463. doi: 10.3389/fmicb.2020.621463 (PMC7900410; doi:10.3389/fmicb.2020.621463)
Supplement: Supplementary file 1 [file Data_Sheet_1.PDF]

Supplementary data

Supplementary Table 1. Synonymous changes in 40F-p8 ORFs related to 56/74

		EXPRESSION HOST ORGANISMS			
**RNA segment**	**residue position**	**MOUSE**	**HUMAN**	**SHEEP**	**AEDES alb.**
L	60	0.53	0.47	0.49	1.16
	126	0.77	0.83	0.64	0.32
	368	0.81	1.04	1.45	0.41
	913	0.67	0.70	0.51	0.60
	1364	0.20	0.17	0.07	0.21
	1658	0.63	0.73	0.38	0.41
	1669	0.25	0.24	0.17	0.39
	1720	0.63	0.73	0.38	0.41
	1725	1.58	1.40	2.63	2.44
	1737	0.77	0.83	0.64	0.32
M	232(Gn)	0.34	0.34	0.40	0.43
	439(Gn)	1.47	1.51	1.03	0.98
	574(Gn)	0.20	0.17	0.07	0.21
	768(Gc)	0.63	0.61	0.39	0.73
	1113(Gc)	0.71	0.67	0.25	0.36
S	30(NSs)	0.97	1.01	0.78	1.40
	188(NSs)	0.67	0.71	0.48	1.24
	234(N)	0.68	0.75	0.69	0.68
	3(N)	0.75	0.86	0.33	0.47

Ratio between genetic codon frequencies in the indicated expression host organisms. Ratio between frequencies per thousand corresponding to codon present in the virus mutant and

codon present in the parental virus, according to https://www.genscript.com/tools/codon-frequency-table, https://www.kazusa.or.jp/codon/. For ratios **below 0.5** the nucleotide change is considered unfavorable in terms of codon usage (shadow).

Supplementary Table 2. List of primers used in this study (RT-PCR and/or sequencing).

Oligo name	sequence (5′-3′)	nt positions[a] (L segment)	orientation
L seg 5′ end	ACACAAAGGCGCCCAATCATGGATTCTATA	1-30	antigenomic
716fwd	CAGTCACAGAATCTGTG	716-732	antigenomic
L-fwd 1028ag	ATGGCAAAGATCTGTGC	1028-1044	antigenomic
L-rev 2300g	AAGTAACCATTGTAACAACA	2300-2281	genomic
RdRp central-fwd	GCTATCCAGAAGTTTGAGGATTG	2701-2723	antigenomic
L-seg-fwd[b,d]	TTCTTTGCTTCTGATACCCTCTG	2872–2894	antigenomic
L-seg-rev[b,d]	GTTCCACTTCCTTGCATCATCTG	3006-2984	genomic
RdRp central-rev	CTGATTTGCAGAAGCTATATGCT	3960-3938	genomic
3817 fwd	GAGTATAAGAAGGCAGT	3817-3833	antigenomic
4553 fwd	TCACTATCTTTAATGAG	4553-4569	antigenomic
5455 fwd	CCATTTGGATGCCCAGTTTATAT	5455-5477	antigenomic
5583 rev	CTGATGAGAATGGTGCT	5583-5567	genomic
Q3′25nts	TTGTAGCACTATGCTAGTATCAGAA	6385-6361	genomic

Oligo name	sequence (5′-3′)	nt positions[a]	orientation

		(M segment)	
(-2)RTsm1	GTTTTATTAACAATTCTAACCTCGGTT	27-53	antigenomic
MRV1ag[c]	CAAATGACTACCAGTCAGC	772-790	antigenomic
RTsm2	CAAGTGAACAGGGAAATAGGATGG	1953-1976	antigenomic
sm2	GAACATGCTGATGCATATGAGACA	2095-2072	genomic
sm3	GACCCATCCTTATTGTGGGCAGAG	3223-3200	genomic
sm4	ATCTGGCAAAAGACGATTACGC	3838-3817	genomic
EM-rev[d]	CACAAAGACCGGTGCAAC	3884-3867	genomic
EM-fwd[d]	GAATCAACAGTTGTGAATCC	3405-3424	antigenomic

**Oligo name**	**sequence (5′-3′)**	**nt positions[a] (S segment)**	**orientation**
NS0g	ACACAAAGACCCCCTAGTG	1-19	genomic
NS2g[e]	GATTTGCAGAGTGGTCGTC	62-80	genomic
revS[d]	CGGACTTGGAGACTTTGCATCA	241-262	genomic
fwdS[d]	TTCTTTCAGATTGGGGAACCTTGT	36-338	antigenomic
NSca[e]	CCTTAACCTCTAATCAAC	841-824	antigenomic
ss1	AGCCACTTAGGCTGCTGTCTTGT	908-930	genomic
RTss1	CTATTACAATAATGGACAACTATCAAGAGC	1662-1633	antigenomic
NP0ag	ACACAAAGCTCCCTAGAGATA	1689-1669	antigenomic

[a]Nucleotide positions according to RVFV MP12 sequence (GB accession: DQ380208.1) [b] Busquets et al. Vector Borne Zoonotic Dis. 2010 10(7):689-96.[c]Takehara et al Virology. 1989 169(2):452-7; [d]Mwaengo et al. Virus Res. 2012 169(1):137-43; [e]Sall et al. Clin Diagn Lab Immunol. 2002 9(3):713-5

Supplementary table 3. List of primers used for RACE

**Oligo name**	**sequence 5′-3′**	**Positions[a] (segment)**	**orientation**

Lseg cRNA	AGTTTTGATAATCATGTC	366-349	genomic
Lseg vRNA	CTTTTGATACATCTGA	6011-6026	antigenomic
Mseg cRNA	GTTTCTCTCCCTATTTG	409-393	genomic
Mseg vRNA	TCCTCCTTATATATCTTG	3544-3561	antigenomic
Sseg cRNA	TATTAGATCAATAAGTCT	313-296	antigenomic
Sseg vRNA	TCTTTCGACATTTTCAT	1419-1435	genomic

[a] nt positions according to the strain 56/74 sequence elucidated in this work (GB annotation pending)
