# Supplementary material for: A Hyper-Attenuated Variant of Rift Valley Fever Virus Generated by a Mutagenic Drug (Favipiravir) Unveils Potential Virulence Markers
Source: Front Microbiol. 2021 Feb 9;11:621463. doi: 10.3389/fmicb.2020.621463 (PMC7900410; doi:10.3389/fmicb.2020.621463)
Supplement: Supplementary file 2 [file Data_Sheet_2.PDF]

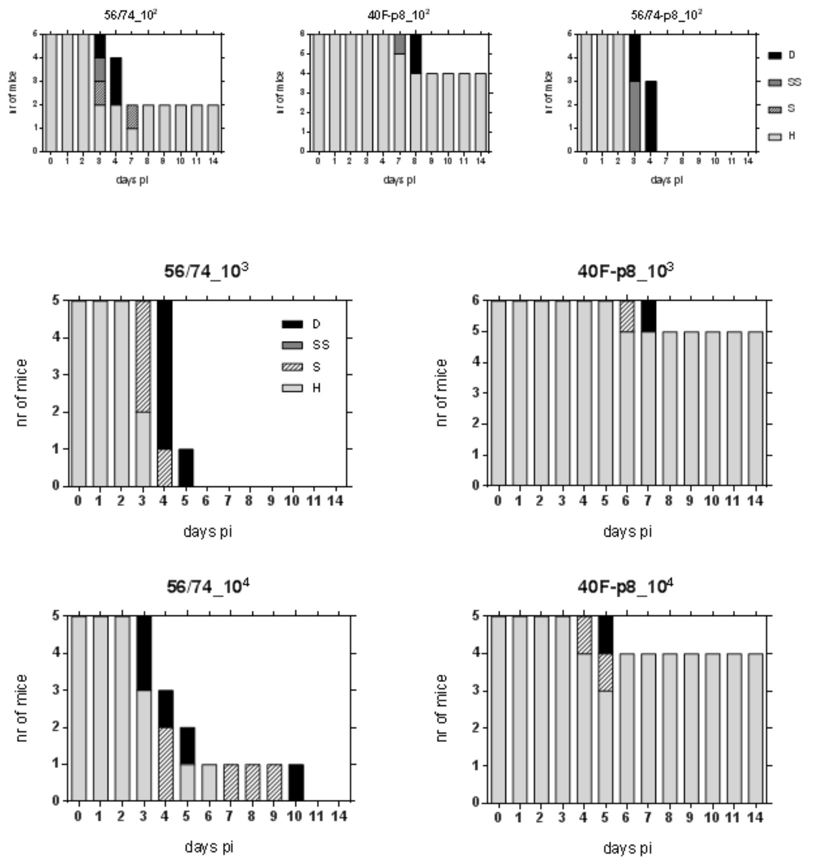

Supplemental Figure S1. Morbidity of A129 mice (IFNAR-/-) upon challenge at the indicated pfu/mice. The graphs represent the clinical status of each mouse: H (healthy), light grey bars; S (signs of disease: ruffled fur, hunched back, watery eye), hatched bars; SS (severe signs: lethargy, paralysis), dark grey bars; D (dead or euthanized): black bars.
